# Toward the Integration of Cyber and Physical Security Monitoring Systems for Critical Infrastructures

**DOI:** 10.3390/s21216970

**Published:** 2021-10-20

**Authors:** Alessandro Fausto, Giovanni Battista Gaggero, Fabio Patrone, Paola Girdinio, Mario Marchese

**Affiliations:** DITEN Department, University of Genoa, 16145 Genoa, Italy; alessandro.fausto@unige.it (A.F.); giovanni.gaggero@edu.unige.it (G.B.G.); paola.girdinio@unige.it (P.G.); mario.marchese@unige.it (M.M.)

**Keywords:** critical infrastructure, cybersecurity, physical security, anomaly detection, machine learning

## Abstract

Critical Infrastructures (CIs) are sensible targets. They could be physically damaged by natural or human actions, causing service disruptions, economic losses, and, in some extreme cases, harm to people. They, therefore, need a high level of protection against possible unintentional and intentional events. In this paper, we show a logical architecture that exploits information from both physical and cybersecurity systems to improve the overall security in a power plant scenario. We propose a Machine Learning (ML)-based anomaly detection approach to detect possible anomaly events by jointly correlating data related to both the physical and cyber domains. The performance evaluation showed encouraging results—obtained by different ML algorithms—which highlights how our proposed approach is able to detect possible abnormal situations that could not have been detected by using only information from either the physical or cyber domain.

## 1. Introduction

Critical Infrastructures (CIs) are “systems that are so vital to a nation that their incapacity or destruction would have a debilitating effect on national security, the economy, or public health and safety” [1]. Traditionally, CI protection has focused on threats coming from the physical world, either environmental phenomena or intentional human actions. This discipline is usually referred to as physical security, which involves the application of resources to the task of protecting physical, human, and intellectual property assets from events such as plunder, theft, or exploitation [2].

Industrial Control Systems (ICSs) play a key role in managing CIs. Some of the most common in use are the Supervisory Control And Data Acquisition (SCADA) systems and the Distributed Control Systems (DCSs), which measure physical quantities such as pressure, current, voltage, and temperature, to monitor CIs within the physical domain. Currently, due to the evolution of technology, networked Information and Communication Technologies (ICTs) pertaining to the cyber domain need to be integrated with these systems. The use of ICTs improves the overall process thanks to a more efficient and dynamic method of exchanging information and managing a higher volume of available data. However, they make ICSs prone to severe cyber threats, endangering process security and the safety of the people involved [3]. Many CIs, such as power systems, that integrate the two domains are thus referred to as Cyber Physical Systems (CPSs) [4], i.e., systems composed of sensors that measure physical quantities and of actuators that operate according to those collected measures. For this reason, these systems are at risk of both physical and remote attacks, increasing their vulnerability to attacks compared to previous isolated system configurations [5,6].

In this paper, we describe a possible first-step approach to integrate existing security monitoring—currently applied only separately to the physical or cyber domain—to achieve a cross-domain. We begin by categorizing industrial security monitoring systems into the three different domains: physical, cyber–physical, and cyber (Section 2). Section 3 proceeds with a discussion about the elements that should be taken into account while designing an integrated security monitoring system and the strategies to establish correlations among different logs. Section 4 analyses the current state-of-the-art regarding integrated security monitoring systems. Then, with Section 5 and Section 6, we propose a logical architecture that is able to detect anomalies by leveraging data received from heterogeneous information sources related to either the physical or cyber domain. The architecture has a tree structure and utilizes Machine Learning (ML) algorithms with different levels of information input, from basic logs to an advanced awareness of the current infrastructure status. We considered as a use case the application of this architecture to an industrial plant, in order to test its feasibility and accuracy. Section 7 and Section 8 show the obtained results and a brief analysis of the collected data, the possible comparison between our approach and others, and some insights on possible future developments that aim to increase the accuracy and robustness of the proposed approach for future real-case applications. Section 9 concludes the paper.

## 2. Security Monitoring

### 2.1. Physical Domain

Information that can be collected from the physical environment is usually very heterogeneous, coming from a variety of different sources.

Physical Access Control Systems (PACSs) control the physical access to a monitored area from the outside and across different zones inside that same area. They are based on Personal Identity Verification (PIV) for people authentication, which is typically based on the common triad of: something you know (such as a password), something you have (such as a smart card), and something you are (such as a fingerprint or other biometric information) [7]. Video surveillance systems increasingly rely on communication systems that can be based on wired or wireless technologies and can use artificial vision techniques for the automatic analysis of recorded videos [8]. Environmental sensor systems include a wide set of possible information sources, and they also often rely on ICT solutions for data transmission. Some examples are: voltage sensors for batteries or Uninterruptible Power Supply (UPS); humidity sensors, to prevent premature aging of equipment; temperature sensors, to detect air conditioning outages, which can be very dangerous for specific devices, such as servers; fluid sensors, to detect water leakages; airflow sensors, to ensure that enough air is flowing through a particular area, preventing hot spots; motion sensors, to detect people’s presence in secure areas with access restrictions; audio sensors, to detect noises, such as breaking glass and alarms.

### 2.2. Cyber–Physical Domain

CPS is an umbrella term that includes different kinds of systems, such as robotics, machine automation, industrial and process control systems, SCADA, the Industrial Internet, and the Internet of Things (IoT) [9]. ICSs, and in particular SCADA systems, log the events related to the industrial process in order to allow operators to supervise the process and guarantee the safety and continuity of the plant operations. In industrial plants, the main goal of an attacker is to modify the physical behavior of the process in order to cause service disruptions and/or damages to devices and even people. If the control network is compromised, an attacker can send fake commands to the actuators, but also act without allowing operators to notice the ongoing attack, as happened with the Stuxnet worm [10]. For these reasons, from a cybersecurity perspective, SCADA often utilizes network and host-based Intrusion Detection Systems (IDSs) and physical-behavior-based anomaly detection algorithms to promptly detect the presence of attacks.

At the moment, a shared standard for logging events in SCADA does not exist. These systems are customizable, and it can be difficult for people, untrained on these systems, to fully understand the events that trigger alarms. This may be a huge problem in correlating and integrating SCADA logs with those of the physical and cyber domains.

### 2.3. Cyber Domain

In ICT systems, almost all devices can generate, store, and send information. Their logs can come from different and numerous sources including firewalls, IDSs, Intrusion Prevention Systems (IPSs), and Virtual Private Networks (VPNs). Complex computer systems collect and analyze this huge amount of information. These system solutions have many different acronyms, such as Enterprise Security Management (ESM), Enterprise Event Management (EEM), Security Information Management (SIM), Security Event Management (SEM), and Security Information Event Management (SIEM). We refer to all of them with the term SIEM.

A SIEM system is generally designed to provide the following set of services [11]:

- Log management: collect, store, and analyze all logs;
- IT regulatory compliance: audit and validate compliance or identify violations of compliance requirements imposed by the organization;
- Event correlation: automatically analyze and correlate data in order to promptly recognize risks;
- Active response: implement countermeasures directly acting from the SIEM system;
- Endpoint security: make adjustments to the node security on the remote system.

**Figure 1:**
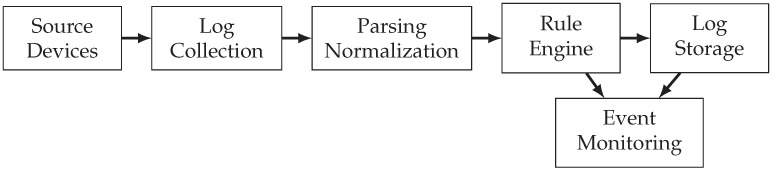
SIEM system processes.

It can be divided into six pieces or processes, as shown in Figure 1.

However, different vendors may produce the devices that generate the input data for the SIEM system. Their data are usually saved in different and proprietary formats. Even the way that events are reported to upstream logging server functions may not be universal [12]. This can create incompatibility when analyzing together data from different sources. Some standards address this issue. In fact, an interesting research field regards the correlation strategies used by the rule engine. While some attack patterns can be easily detected by using simple rules, more complex attacks may require more sophisticated approaches—which may use the capabilities of ML algorithms–to be detected.

### 2.4. Human–Machine Interface

Once the system has collected logs, it must prioritize the information and alert analysts about potential issues. These systems can be used both for online monitoring—allowing a prompt response to possible threats—and for a posteriori analysis, offering support in forensics investigations. The process of security is deter–detect–delay–respond. An integrated SIEM system should have a fundamental role in the last three stages. Visual analytics can be of great help for operators in managing the network. Nevertheless, the need for automatic approaches arises with the increase of the system size and complexity. Big data offer great possibilities for the analysis of complex systems. For this reason, correlation engines in IDS and SIEM systems can make great use of ML technologies.

## 3. Design of Integrated Siem

To start designing log management systems, there are three basic questions to keep in mind: What kind of information logs are useful? How many logs have to be measured? How long do logs have to be stored?

Concerning the first question, information logs can be produced by any device on a network. Each log source can usually be tuned to provide a record of multiple kinds of information with different detail levels depending on the use. Concerning the other two questions, the possible answers strongly depend on the implemented correlation logic. The correlation among different logs may be unexpected, but fundamental to improve security monitoring. Observing the same log over time may reveal important details, as well as establish a correlation among different logs measured at shifted time instants. Within an extended time range, the correlation engine could employ different strategies to exploit the stored data in order to identify possible risk situations with higher accuracy.

Once all heterogeneous data—generated by multiple sources within a monitored area—have been collected, possible strategies to manage the complexity and identify dangerous situations (threats) have to be properly designed.

### 3.1. Correlation by Logic

Even the occurrence of regular events may pose a threat when correlated together in the following ways:

- **Correlation by physical area:** In the case of a delimited physical area, a possible risk situation can arise when, for example, one correlates the presence of a person with the use of a PC device. If the PC IP address were active and accessing an internal and protected data subnet, but the corresponding user was not yet present on premises, then that activity could be a potential threat;
- **Correlation by person:** Tracking the activity of a person within a monitored area can be very useful to prevent threats, even if it involves different physical and ICT log systems. Since most humans are habitual, algorithms can notice unusual behaviors that can be labeled as an anomaly. Additionally, correlating people’s physical and remote accesses can reveal malicious actions. For example, a person that simultaneously accesses a PC both physically and remotely is a suspicious event;
- **Correlation by time:** Two events that occur within a limited time interval could be the symptom of a causal correlation between them. A simple example is the activity of port scanning within the network followed by multiple failed login attempts. However, properly identifying which events should be causally correlated and, in case, which time frame is appropriate, may not be an easy task.

### 3.2. Correlation by Methods

Three main possible solutions to establish correlation are:

- **Visual analytics:** an approach that involves the design of proper Human–Machine Interfaces (HMIs) to visually highlight correlations among collected data to human operators. They typically involve different windows and different graphical solutions;
- **Rules:** Fixed rules can be set to check simple conditions. SIEM systems can implement automatic algorithms based on “if/if then else” sentences coming, for example, from corporate policies or simple potential risk situations identified a priori;
- **Machine learning:** In case a set of basic rules is not enough to properly depict the overall set of possible anomaly situations, ML-based anomaly detection solutions can be employed to learn the specific patterns and habits of employees without explicitly declaring which behaviors are considered abnormal. They can then autonomously understand if there are any anomalies.

## 4. State-of-the-Art on Siem

Within the cybersecurity literature, we can find some research related to the correlation issue. An example of traditional security monitoring technologies are network IDSs [13] and host IDSs [14]. These technologies make large use of ML techniques [15]. Security monitoring of ICSs increasingly takes into account heterogeneous sources of information [16]; for this reason, the so-called physics-based attack detection algorithms [17] are an increasingly promising field of research, relevant also for microgrids [18] and Distributed Energy Resources (DERs) [19]. Particularly interesting in the field of ICS security are anomaly detection (or novelty detection) algorithms [20,21,22], which could be applied in a variety of cyber–physical scenarios.

Some papers focus on the correlation of events within SIEM systems to reduce network complexity. They investigate strategies for preprocessing alarm events in order to reduce the number of displayed alarms, thus simplifying the system for human operators. An overview of the most popular SIEM tools and open-source rule-based correlation engines (including IBM QRadar, HP ArcSight, Splunk, and LogRhythm) was presented in [23], which compared the engine correlation mechanisms and classified them into similarity-based, knowledge-based, and statistical correlation. The authors of [24] proposed two novel alert correlation approaches for SIEM systems: enforcement-based correlation, which aims at classifying all possible countermeasures and their associated policy enforcement points to implement the security rule as a defense mechanism; metric-based correlation, which aims at deriving correlation rules from information security indicators to allow the analysis and evaluation of the SIEM effectiveness.

However, only a few works focus on the correlation of heterogeneous events for security reasons. One of the most challenging goals is to discover complex attack patterns through the combination of physical and cyber events. Some preliminary approaches for the integration of heterogeneous data sources and the correlation of apparently disparate events for protection against cyber–physical attacks were reported in [25]. A framework for event collection and correlation that can process and analyze heterogeneous data through event pattern detectors—and integrate them into the open-source SIEM OSSIM—was proposed in [26]. The authors of [27] addressed the issue of physical security information management and security information and event management integration by using the IBM SIEM QRadar as a platform. The authors of [28] presented another framework, called synERGY, for cross-layer anomaly detection based on ML techniques, in order to enable the early discovery of both cyber and physical attacks that may impact the cyber–physical system.

The discussed works presented interesting solutions for the implementation of a security monitoring system by using already developed and off-the-shelf SIEM infrastructures. Nonetheless, the correlation strategies of heterogeneous events for security reasons is still an open issue, as well as the techniques and algorithms that can allow exploiting this correlation. The solutions proposed in the state-of-the-art cover only a small portion of the possible use cases and are difficult to compare with each other due to the lack of shared use cases and datasets. Our proposed solution begins covering this gap and consists of an innovative approach based on the definition of a state vector as a representation of the considered industrial plant use case. The proposed logical architecture involves the extraction of useful information from the state vector that can then be processed by ML algorithms to detect anomalies.

## 5. Industrial Plant Use Case

**Figure 2:**
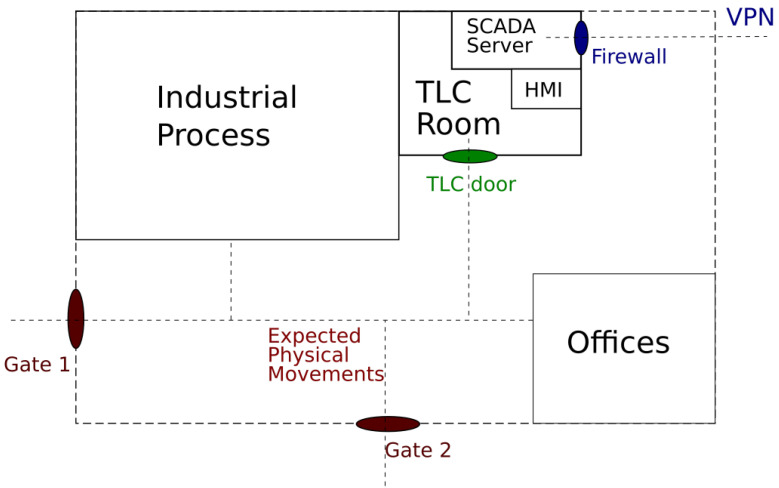
Schematic representation of the considered scenario.

As a first step, let us consider a generic industrial plant, e.g., a power plant, which is located within a fenced area. Its schematic representation is shown in Figure 2.

The industrial process is controlled by a SCADA system whose servers are located in a dedicated room, which we call the TLC room. It also contains the HMI for the operators. All the employees are identified at the gates of the industrial plant, and only a portion of them are allowed to access the TLC room. Therefore, the plant implements two main physical log systems: the first one manages the accesses to the plant, and the second one manages those to the TLC room. The SCADA system is connected to the TCP/IP-based enterprise network through a firewall. The SCADA server can be reached from the outside only by using a Virtual Private Network (VPN). This situation is quite common for industrial plants that allow authorized users to connect remotely to a SCADA server for assistance and maintenance. The VPN system logs all the accesses and some information about the exchanged traffic.

In the considered scenario, the main target of attacks is the SCADA system. The attacks aim to interfere with the normal industrial process to create economic damages, service disruptions, and damages to devices and even people. As we mentioned, the SCADA server can be accessed both physically and remotely. An attack against the control system can therefore be carried out by physically reaching the control device—for example, by stealing the badge of a person authorized to access the plant and the TLC room—or remotely, by exploiting vulnerabilities of the cyber defense system or by obtaining remote access credentials. An attack can also be carried out by a combination of physical and remote strategies. An example is acting through abandoned USB pen drives waiting to be picked up by an inadvertent employee, who will plug them into work PCs or possibly in the TLC room. One of the most dangerous threats is represented by insiders, i.e., people that are normally authorized to access the plant but decide to “switch sides”. For these many reasons, there is a huge variety of potential risk situations that have to be considered in an effective use case scenario.

## 6. Proposed Approach

### 6.1. General Description

Due to the complexity of taking into account such diverse data simultaneously, the proposed approach is based on the idea of decomposing the complexity in different analysis levels. We designed a conceptual architecture that is able to manage data coming from an industrial plant and is also scalable, to allow possible further integration of additional log systems. In order to do this, the output of each log system is preprocessed and feeds multiple ML algorithms that act in parallel. Each of them is tasked with detecting different kinds of anomalies depending on the related subset of log systems. Subsequently, the architecture analyses the output of these algorithms to detect potential ongoing attacks.

In order to allow better understanding of the approach description, we define the following terms:

- **Log**: any type of information, such as raw text lines or numbers, from any considered type of log devices;
- **Event**: the result of the preprocessing of one or more logs that identifies an occurrence within a single log system;
- **Anomaly**: the output of a single ML algorithm that can take into account events from one or more log systems;
- **Alarm**: the signaling of a risk situation within a monitored area due to the contemporary presence of one or multiple anomalies that identify a potential ongoing attack.

**Figure 3:**
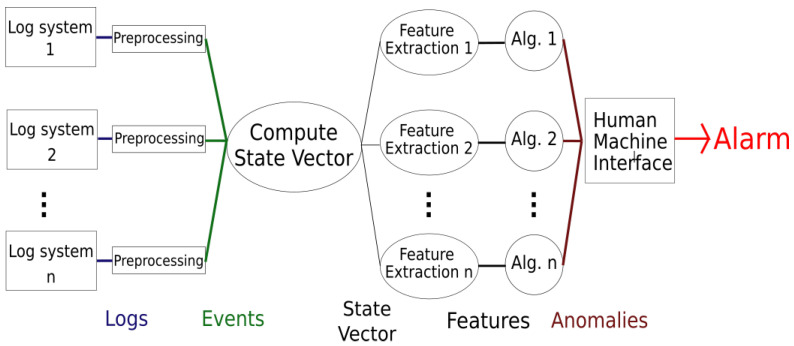
Logical architecture of the proposed approach.

The logical architecture of the proposed approach is shown in Figure 3.

The proposed solution works in real-time. Each time a log is collected by a log system, it is sent to a related preprocessing block. This phase is particularly important to mitigate the effects of errors—which can occur depending on the used technology—during the log phase. For example, many systems for access control based on RFID register spurious events. The preprocessing phase is fundamental to transform raw logs into events.

In order to take into account all the events that occur within the plant, the proposed solution builds a representation of the current working conditions of the whole plant, which we call the state vector. The state vector stores all the considered information, such as the physical or remote accesses of each employee. Each time the system processes a new event, it updates the state vector so that it is a concise real-time representation of the plant.

The whole state vector cannot directly feed the ML algorithms. The events that contribute to the creation of the state vector typically contain much information, and only some of it is useful for the considered task. The specific information—called features—is extracted from the state vector and used to feed the ML algorithms. Multiple algorithms run in parallel and analyze different subsets of features. Thus, every time the system processes a new event, it not only updates the state vector, but extracts a set of features—grouped in the feature vector—and sends it to the related ML algorithms. Different types of events can trigger different ML algorithms. ML algorithms detect specific abnormal situations—if any—that are then signaled to the human operators through a proper HMI.

Finally, human operators have all the information to decide if detected anomalies are false alarms or if they could represent a real threat, in which case they will start the required countermeasures to properly manage it.

### 6.2. Setup of the Proposed Approach

**Figure 4:**
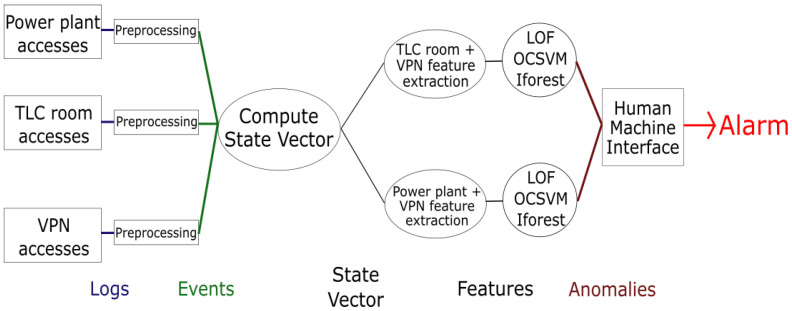
Implementation of the proposed architecture.

To move forward to the proposed architecture implementation, we considered three different log systems: the physical access to the power plant through the perimeter gate, the physical access to the TLC room, and the remote access via the VPN to the SCADA server. We also considered two parallel ML algorithms: the first one focuses on the possible anomalies related to the TLC room access, while the second one centers on possible anomalies related to the power plant access. The overall implementation of the proposed architecture is shown in Figure 4. The code was in Python, and the modules are detailed below.

The first module of the algorithm preprocesses the input data. In this case, we used a three-month period log of monitoring systems from a real power plant. Different external companies have set up and managed these log systems, which were not designed to allow generated data correlation. The access log system of the power plant gate identifies authorized users by first and last name or by car registration number; the access log system of the TLC room identifies authorized users by first and last name or by employee ID; the access log system of the VPN identifies authorized users by an identifier, which can be related to a single person or to a company. Since identifying the same person through these three log systems may not be straightforward, we preprocessed the overall log dataset to solve this user identification issue and to remove some log errors.

We define some specific features, which are reported and described in Table 1 and Table 2.

We considered three widespread anomaly detection algorithms: Local Outlier Factory (LOF), Isolation Forest (IForest), and One-Class Support Vector Machine (OCSVM). LOF is based on the concept of local density, where locality is given by the k nearest neighbors whose distance is used to estimate the density. It is possible to identify regions of similar density that have a substantially lower density than that of their neighbors by comparing the local density of an object to the local densities of its neighbors, considered as outliers [29]. Isolation forest is an algorithm based on decision trees that explicitly identify anomalies instead of profiling normal data points. Anomalous instances in a dataset can be more easily separated from the rest of the samples than normal points by using the isolation forest algorithm. To isolate a data point, the algorithm recursively generates partitions of the sample by randomly selecting an attribute and then randomly selecting a split value for the attribute between the minimum and maximum values allowed for that attribute [30]. OCSVM is a natural extension of the Support Vector Machine (SVM) algorithm in the case of unlabeled data. It consists of a discriminant function that identifies a small region where the density of the feature values is highest and sets a value of +1, while it assigns to the remaining outside area a−1 [31]. All the described algorithms were implemented by using the SciKit Learn library [32]. Additional details about the technical implementation of the described setup can be found in Appendix A.

To train the ML algorithms, we used a dataset that does not contain any event that should be classified as anomalous. To test the algorithms, we used a test set containing different possible anomalies.

It is necessary to highlight that, unlike other types of analyses in the field of cybersecurity —such as malware analyses by network traffic, in which the portion of traffic related to the malware is clearly defined—in the considered scenario, there are no past examples of complex cyber–physical attacks, and consequently, it is not possible to use an already available attack scenario. To overcome this limitation, we imagined five kinds of possible anomalous events that deviate significantly from normal log patterns and that can represent a threat:

- Access of an employee at an unusual time and/or day to the TLC room;
- VPN access from users already physically present in the power plant or the TLC room;
- Multiple accesses of the same employee to the TLC room;
- Multiple accesses of the same employee to the power plant;
- Access of an employee to the TLC room without previous access to the power plant.

In the field of industrial system cybersecurity, there are very few event descriptions available (and even less for the public domain) regarding complex cyber–physical attacks. For this reason, the performance evaluation shown in this paper is not related to the ability of the proposed solution to detect ongoing attacks, but to detect possible situations of potential risk.

The test set was composed of 97.7% normal events (negative events) and 2.3% anomalies (positive events).

## 7. Performance Evaluation

The results obtained by using each of the three considered ML algorithms are shown through the confusion matrices reported in Table 3, Table 4 and Table 5 for LOF, isolation forest, and OCSVM, respectively.

LOF detects 100% anomalies and interprets correctly most normal events. On the other hand, isolation forest’s performance is not satisfying. As OVCSM, it has difficulty with detecting anomalies. These behaviors are even clearer by comparing the three ML algorithms through three metrics commonly used in ML, i.e., accuracy, sensitivity, and specificity, which are defined by Equations (1)–(3), respectively:

(1) $$Accuracy=\frac{TP+TN}{TP+TN+FP+FN}$$

(2) $$Sensitivity=\frac{TP}{TP+FN}$$

(3) $$Specificity=\frac{TN}{TN+FP}$$

where TP: True Positive, TN: True Negative, FP: False Positive, and FN: False Negative.

The obtained results are reported in Table 6.

## 8. Discussion

After the testing sessions, our observation was that LOF offers the best performance. Even if the difference does not seem so significant by looking at the accuracy results, it actually is. Considering the unbalanced number of positive and negative samples in the test set, even a system that detects no anomalies obtains an accuracy of 97.7%, i.e., the percentage of negative samples in the test set. Isolation forest and OCSVM are unable to efficiently recognize anomalies. They also misclassify some negative events and identify them as anomalies. The sensitivity and specificity results emphasize this. We also tried to properly set the configuration parameters of both algorithms by analyzing if their setup significantly affects the obtained performance. We found out that higher sensitivity could have been obtained by using different parameter configurations for both isolation forest and OCSVM, but with a consequent lower specificity and vice versa, so without improving the overall performance.

The results are hardly comparable with the works reported in Section 4. The works in the literature significantly differ both from the use case scenario and for the type of data considered. However, some considerations can still be made. Rule-based approaches are of course useful to detect specific behaviors and working conditions that can be considered dangerous. Nevertheless, they require high customization on the considered environment, which can be extremely variable in terms of physical structure, network architecture, log systems, and user behavior, among other factors. For these reasons, ML approaches can be very helpful to address these issues. The results presented in Section 7 are, although preliminary, really promising. Our proposed system:

- Is able to detect possible anomaly situations or attacks that cannot be detected by the traditional security mechanisms thanks to the joint use of multiple information sources;
- Is more robust against actions that aim to break the security systems (e.g., if stealing a badge may be enough to let a malicious person enter a power plant, it would be less easy if there were multiple security systems to corrupt);
- Offers an automatic tool able to correlate data generated by heterogeneous sources thanks to its ML core, i.e., thanks to ML algorithms able to effectively identify both known and possible unknown anomalies exploiting hidden information inside the typically huge amount of raw data.
- Supports human security officers that could be distracted by the huge amount of available data or by other events taking place within the monitored area.

The main limitation of the present work is certainly the need to simulate datasets related to attacks rather than being able to work with real cases. Since we did not dispose of real data about real attacks in scenarios such as industrial plants, we had to generate the related data attack traces through a simulation environment. That condition is unfortunately common for this type of research since industries will hardly release such types of information. To relieve this limitation, it would be useful to proceed with interviews to power plan operators, allowing researchers to dispose of a set of possible complex attack patterns to test the proposed solutions.

Another interesting future development of this work would be the inclusion of logs belonging to a higher number of different systems that could allow having more accurate information even if, in some cases, the cost could be higher redundancy. For example, from the physical world, data from intelligent camera systems processed with image recognition algorithms could provide useful information to relate to other log systems. In this way, the presence of a person in a room could be related to the data from cameras, the access log through badges, accounts in use on the room’s terminals, and the use of the user’s IP address. Such a system will be more robust to attacks that have to compromise multiple systems to enter into action and keep working undetected.

## 9. Conclusions

Cybersecurity is of primary importance in critical infrastructures. Physical and cybersecurity domains are often still considered separate domains and have not been designed to work together by exchanging data in order to improve their performance. Nonetheless, the two domains are becoming more and more indistinguishable, and this feature should be benefited from.

Very few works focus specifically on the integration of logs belonging both to the physical and cyber domains. Some related works on SIEM correlation rules have been discussed, but the context and types of data differ from the ones considered in our work.

Our proposed approach jointly considers data generated by both physical and cybersecurity systems to detect possible risk situations within a power plant use case. The logical architecture, which has a generic value and could also be applicable in other scenarios, is based on machine learning algorithms for anomaly detection and considers, as the input, events extracted from log files generated by different monitoring systems related to both physical and cybersecurity. Different ML algorithms have been considered to test the proposed solution with encouraging results.

Possible future developments involve the use of data from past attacks of real power plants and an increase of the considered systems and input data to further strengthen the accuracy and robustness of the proposed approach.

## Figures and Tables

**Table 1:** TLC room + VPN access-related features.

Name	Description
Weekday	integer value related to the day of the week when the event occurs (from 0: Monday to 6: Sunday)
Hour	time (in 24 h format) when the event takes place
TLC access error	binary value, which is 1 if the system rejects a person’s access to the TLC room or 0 if the access request is accepted
Direction	binary feature whose value is 1 if the user enters the room or −1 if he/she leaves the room
TLC presence	integer value, which is increased by 1 when the user goes in the TLC room and decreased by 1 if he/she goes out. Normally, it will be 0 or 1. In the case of multiple accesses, i.e., when the log system records two entrances of the same user without recording an exit between the first and the second entrance, this feature takes the value 2. It can take higher values in the case of further consecutive entrances. To avoid keeping an incorrect offset, i.e., to avoid that an anomaly goes on affecting the value of this feature after it has been identified, this value is reset under certain conditions. For example, after two following entrances of the same user, it takes the value 2 for that user, but after a first single exit, the value is reduced to 0 instead of 1.
Power plant presence	integer value, which is increased by 1 when the user enters the power plant and decreased by 1 if he/she leaves the power plant. This feature can behave as the “TLC presence” feature.
VPN presence	integer value, which is increased by 1 when the user obtains remote access to the SCADA server and decreased by 1 if he/she logs out. This feature can behave as the “TLC presence” feature.

**Table 2:** Power plant + VPN access-related features.

Name	Description
Weekday	in Table 1
Hour	in Table 1
Power plant access error	binary value, which is 1 if the system rejects a person’s access to the power plant or 0 if the access request is accepted
Direction	binary feature whose value is 1 if the user enters the power plant or −1 if he/she leaves the power plant
Power plant presence	integer value, which is increased by 1 when the user enters the power plant and decreased by 1 if he/she leaves the power plant. Normally, it will be 0 or 1. In the case of multiple accesses, i.e., when the log system records two entrances of the same user without recording an exit between the first and the second entrance, this feature takes the value 2. It can take higher values in the case of further consecutive entrances. To avoid keeping an incorrect offset, as described in the previous table, this value is reset under certain conditions.
VPN presence	integer value, which is increased by 1 when the user obtains remote access to the SCADA server and decreased by 1 if he/she logs out. This feature can behave as the “power plant presence” feature.

**Table 3:** Confusion matrix: LOF algorithm.

		Predicted	
		Positive	Negative
Real	Positive	2.3%	0%
	Negative	1.9%	95.8%

**Table 4:** Confusion matrix: isolation forest algorithm.

		Predicted	
		Positive	Negative
Real	Positive	1.3%	1%
	Negative	10.2%	87.5%

**Table 5:** Confusion matrix: OCSVM algorithm.

		Predicted	
		Positive	Negative
Real	Positive	1%	1.3%
	Negative	7%	90.7%

**Table 6:** Comparison among the considered ML algorithms.

	LOF	IForest	OCSVM
Accuracy	98.1%	88.7%	91.7%
Sensitivity	100%	57.1%	42.9%
Specificity	98%	89.5%	92.8%

**Figure A1:**
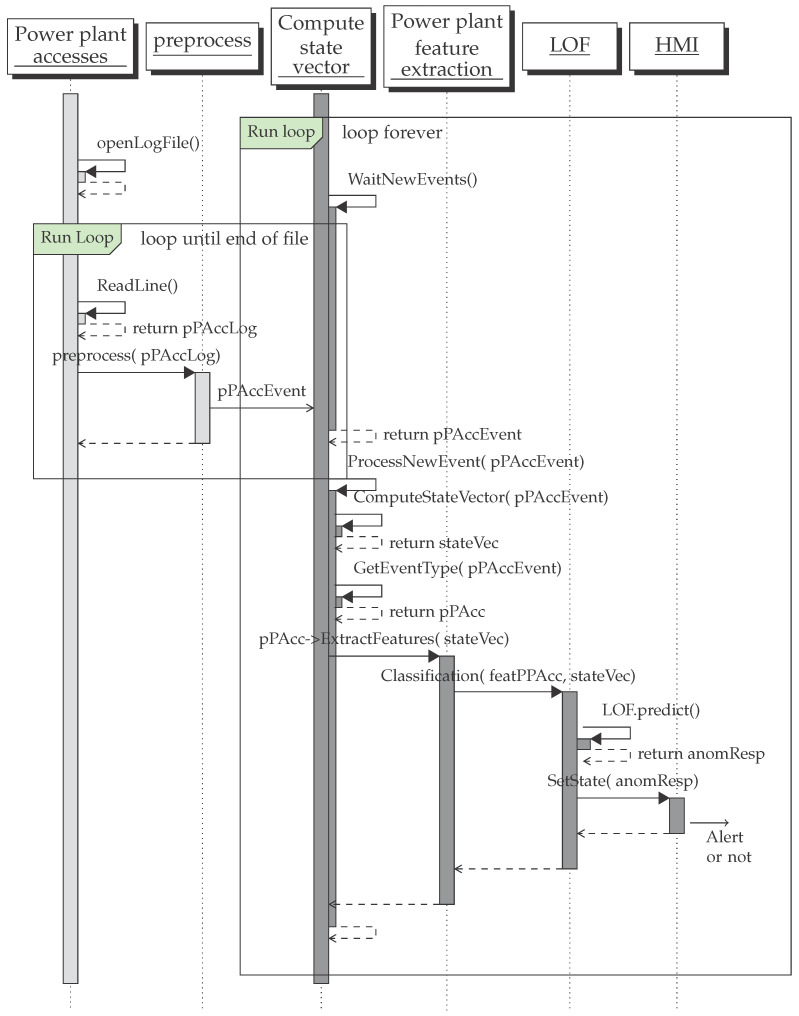
Sequence diagram showing the system operations when a new log (a power plant access log, as an example) is registered.

**Figure A2:**
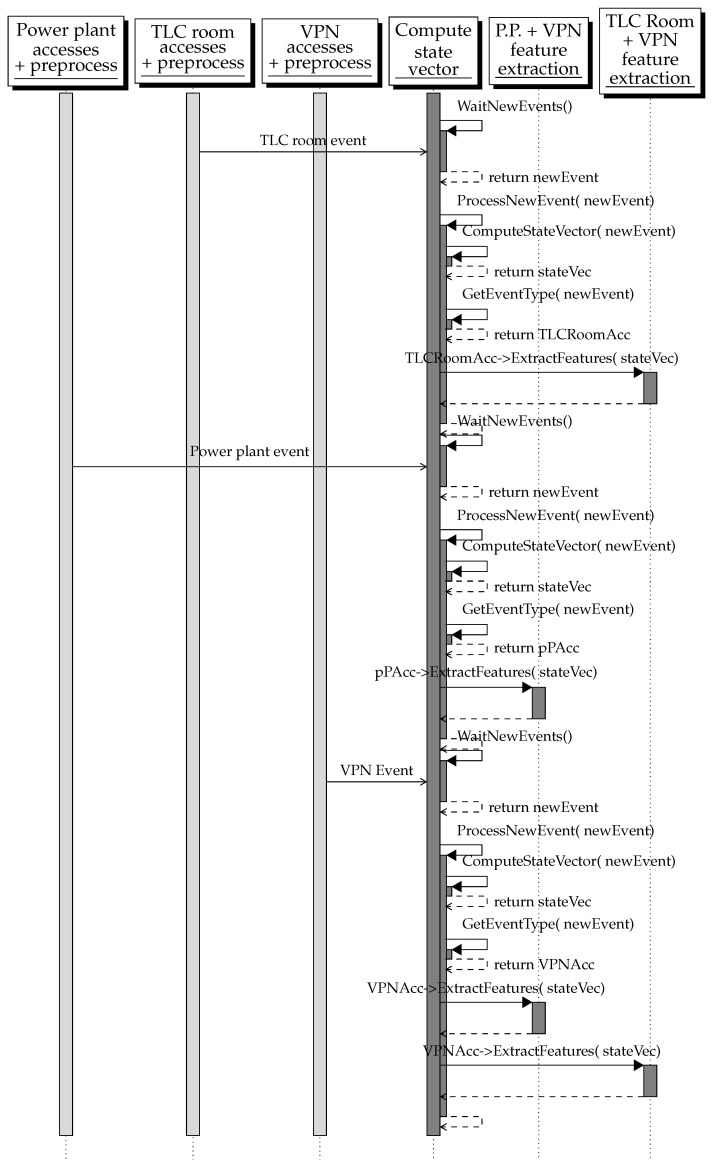
Sequence diagram showing the complete first part of the implemented system with three log sources and two extraction blocks.Handling of parallel events.

## Abbreviations

The following abbreviations are used in this manuscript:

CI	Critical Infrastructure
CPS	Cyber Physical System
DCS	Distributed Control System
DER	Distributed Energy Resource
EEM	Enterprise Event Management
ESM	Enterprise Security Management
FN	False Negative
FP	False Positive
HMI	Human–Machine Interface
ICS	Industrial Control System
ICT	Information and Communication Technology
IDS	Intrusion Detection System
IoT	Internet of Things
IPS	Intrusion Prevention System
LOF	Local Outlier Factory
ML	Machine Learning
OCSVM	One-Class Support Vector Machine
OT	Operation Technologies
PACS	Physical Access Control System
PIV	Personal Identity Verification
SCADA	Supervisory Control And Data Acquisition
SEM	Security Event Management
SIEM	Security Information Event Management
SIM	Security Information Management
TN	True Negative
TP	True Positive
UPS	Uninterruptible Power Supply
VPN	Virtual Private Network

## Appendix A

In this Appendix, we included two sequence diagrams that explain further in detail the software implementation of our proposed approach and the interactions among the blocks in Figure 4. Figure A1 shows the complete end-to-end interactions among the components, considering only one of the log sources and the related feature extraction block, while Figure A2 shows the complete view with all three available information sources and the two feature extraction blocks.

Looking at Figure A1, one can notice that a state vector computation is triggered every time a new log is registered (in this case, a new power plant access log). This action triggers in turn the extraction of a new feature vector from the related feature extraction block. A following classification takes place in order to identify if the current system state is normal or not by using one of the considered ML algorithms (in this case, the LOF algorithm). If an anomaly is detected, an alarm is triggered. If not, the system comes back to the idle state, waiting for the following log. If a log is received while the system is already processing the previous log, the related event is queued within the compute state vector block, waiting to be considered for the following state vector computation.

Looking at Figure A2, one can notice that both power plant access logs and TLC room access logs trigger a state vector computation and a following feature extraction only of the related feature extraction block. VPN access logs, instead, trigger the state vector computation and a following feature extraction in both implemented feature extraction blocks, considering that some anomalies, such as the considered “unusual VPN access from users already physically present in the power plant or the TLC room”, correlate the VPN access data with both power plant and TLC room access data.
